# Precise safety pharmacology studies of lapatinib for onco-cardiology assessed using *in vivo* canine models

**DOI:** 10.1038/s41598-020-57601-x

**Published:** 2020-01-20

**Authors:** Kentaro Ando, Takeshi Wada, Xin Cao

**Affiliations:** 1 Department of Pharmacology, Faculty of Pharmacy, Chiba Institute of Science, 15-8 Shiomi-cho, Choshi, Chiba 288-0025 Japan; 2 Department of Cardiology, Juntendo University Urayasu Hospital, 2-1-1 Tomioka, Urayasu, Chiba 279-0021 Japan; 3 Acupuncture and Tuina School/Third Teaching Hospital, Chengdu University of Traditional Chinese Medicine, No. 37 Shierqiao Road, Jinniu District, Chengdu, Sichuan 610075 China

**Keywords:** Electrocardiography - EKG, Mechanism of action, Pharmacodynamics, Hypertension, Breast cancer

## Abstract

Cancer chemotherapies have improved prognosis in cancer patients, resulting in a large and rapidly increasing number of cancer survivors. “Onco-cardiology” or “cardio-oncology” is a new discipline for addressing the unanticipated cardiac side effects of newly developed cancer drugs. Lapatinib, a tyrosine kinase inhibitor suppressing the epidermal growth factor receptor and ErbB2, has been used in advanced or metastatic breast cancer treatment. Reportedly, lapatinib has induced cardiovascular adverse events including QT-interval prolongation and heart failure. However, they have not been predicted by preclinical studies. Hence, a new method to assess the tyrosine kinase inhibitor-induced adverse effects needs to be established. Here, we intravenously administered lapatinib to halothane-anaesthetised dogs, evaluating cardiohemodynamic, electrophysiological, and echocardiographic profiles for pharmacological safety assessments. We intravenously administered lapatinib to chronic atrioventricular block beagle dogs to assess its proarrhythmic potential. The therapeutic concentration of lapatinib significantly increased total peripheral vascular resistance, QT, QTc, monophasic action potential (MAP)_90(sinus),_ MAP_90(CL400)_, effective refractory period, and plasma concentration of cardiac troponin I (cTnI), suggesting that lapatinib prolonged the ventricular repolarization without inducing lethal ventricular arrhythmia. Careful monitoring of plasma cTnI concentration and an electrocardiogram could be supportive biomarkers, predicting the onset of lapatinib-induced cardiovascular adverse events.

## Introduction

Advancements in new cancer chemotherapies have been improving the prognosis of cancer patients, resulting in a large and rapidly increasing number of cancer survivors. However, their life after cancer is often hindered by unexpected adverse events resulting from anti-cancer therapy. For example, a certain type of tyrosine kinase inhibitors has yielded unanticipated cardiac adverse events in frequency and severity. A new discipline called “onco-cardiology” or “cardio-oncology”, aimed at monitoring the cardiovascular safety of antitumour therapies, has evolved over the past decade in the clinical setting. Although popularly perceived as a clinical discipline that brings oncologists and cardiologists working together, cardio-oncology is, in fact, a pharmacology-oriented translational discipline^1^.

The ICH S9 guideline provides information for pharmaceuticals that are intended to treat cancer in patients with serious and life threatening malignancies^2^. Generally, safety pharmacology studies are not carried out minutely in common anticancer drugs during preclinical studies, since this guideline describes that in the absence of a specific risk, the studies will not be called for to support clinical trials or for marketing^2^. On the contrary, evaluation of the adverse pharmacodynamic and/or pathophysiological effects of a drug, observed during clinical studies, is one of objectives of the safety pharmacology studies^3^. The implementation of precise safety pharmacology studies according to follow-up studies for the safety pharmacology core battery may alter the unexpected adverse events to expected ones, leading to contribute to provide a better quality of life in cancer survivors^3^.

Tyrosine kinase inhibitors have been widely used for the treatment of various types of cancers. Lapatinib is a tyrosine kinase inhibitor that suppresses both the epidermal growth factor receptor (EGFR or ErbB1) and ErbB2 (HER2), for the treating patients with advanced or metastatic breast cancer^4^. This drug has been used in the treatment of patients with advanced or metastatic breast cancer who have received prior therapy including anthracyclines, taxane, and trastuzumab. Diarrhoea is one of the most common adverse events of lapatinib, but cardiotoxicity should be monitored as a side effect^4–6^. The overall incidence of cardiac adverse events with lapatinib has been reported to be 2.70% (95% confidence interval: 1.60–4.50%), including heart failure in patients with breast cancer and other HER2-positive cancers^7^. Importantly, the drug has been also reported to prolong QT interval in the clinical use^7^ and has been shown to inhibit the human *ether-à-go-go-related* gene (hERG) current in a concentration-dependent manner^8^. However, this potential for QT-interval prolongation has not been indicated in preclinical telemetry studies using dogs^4^. Lapatinib has slightly increased mean systolic, mean diastolic, and mean arterial pressure in telemetered dogs at single oral doses ≥150 mg/kg 6 to 14 h after dosing^4^. On the contrary, no significant changes in blood pressure have occurred in patients administered lapatinib^4^.

To the best of knowledge, safety pharmacological assessments of lapatinib evaluating onco-cardiology have not been precisely investigated in non-clinical studies. There are no established methods to precisely predict the lapatinib-induced adverse effect. Hence, in this study, we simultaneously assessed the cardiohemodynamic electrophysiological, and echocardiographic profiles of lapatinib using the halothane-anaesthetised canine model. Furthermore, we assessed the proarrhythmic effects using the chronic atrioventricular block model in dogs. Notably, lapatinib binds to their ErbB2 with the similar potency of the human receptor based on sequence considerations^4^. In addition, we evaluated some blood biochemical markers to predict its cardiotoxicities. These studies would be translational research to clarify the cardiovascular adverse events in clinical practice.

## Results

### Experiment 1: Effects of lapatinib on the halothane-anaesthetised dogs

No animals demonstrated any lethal ventricular arrhythmia or hemodynamic collapse, leading to the animals’ death during the experiment.

### Effects on the cardiohemodynamic variables

**Figure 1:**
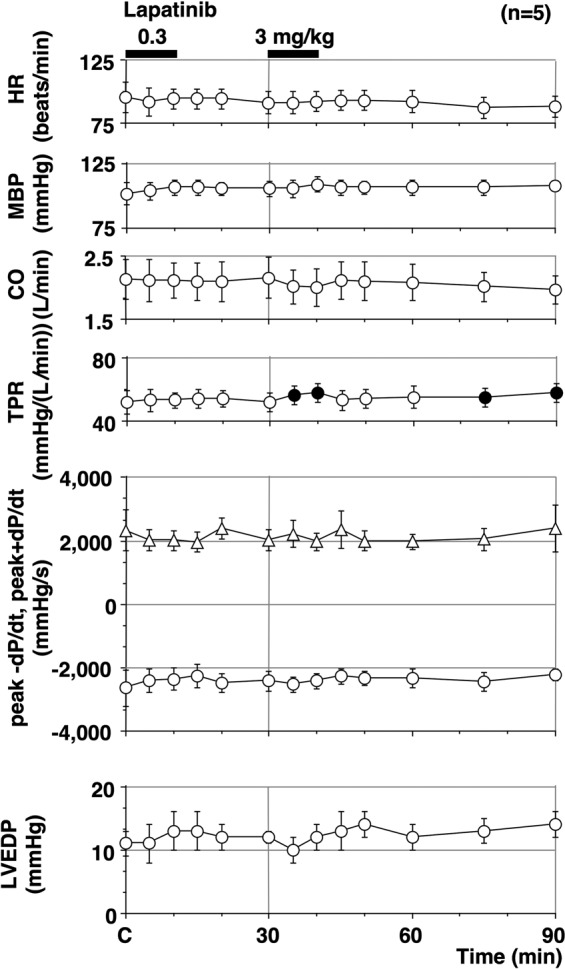
Time courses of change in the heart rate (HR), mean blood pressure (MBP), cardiac output (CO), total peripheral vascular resistance (TPR), maximum upstroke velocity of the left ventricular pressure (peak +d*P*/d*t*), maximum downstroke velocity of the left ventricular pressure (peak −d*P*/d*t*) and left ventricular end-diastolic pressure (LVEDP) after the administration of lapatinib. Data are presented as mean ± SEM (n = 5). Closed symbols represent significant differences from the corresponding pre-drug basal control values (C) by p < 0.05.

The time courses of changes in the heart rate, mean blood pressure, cardiac output, total peripheral vascular resistance, peak + d*P*/d*t*, peak −d*P*/d*t*, and the left ventricular end-diastolic pressures (LVEDP) are summarised in Fig. 1 (n = 5). The pre-drug control values (C) were 95 ± 12 beats/min, 102 ± 9 mmHg, 2.1 ± 0.3 L/min, 51.7 ± 7.2 mmHg/(L/min), 2,328 ± 640 mmHg/s, −2,646 ± 582 mmHg/s, and 11 ± 2 mmHg, respectively. Lapatinib significantly increased the total peripheral vascular resistance for 5–10 and 45–60 min after the high dose. No significant changes were detected in the other variables.

### Effects on the electrocardiogram during the sinus rhythm

**Figure 2:**
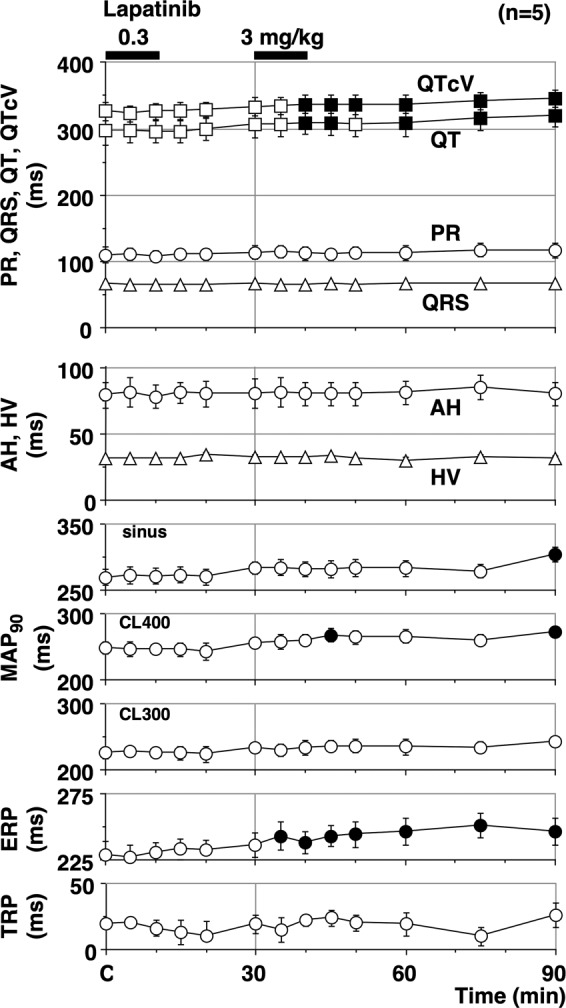
Time courses of changes in the PR interval (circles), QRS width (triangles), QT interval (squares) and QTcV (squares), atrio-His interval (AH; circles), His-ventricular interval (HV; triangles); the MAP_90_ at sinus rhythm, pacing cycle length of 400 ms (MAP_90(CL400)_) and 300 ms (MAP_90(CL300)_); the effective refractory period of the right ventricle (ERP) and terminal repolarization period (TRP) after the administration of lapatinib. QT interval was corrected with Van de Water’s formula. Data are presented as mean ± SEM (n = 5). Closed symbols represent significant differences from the corresponding pre-drug basal control values (C) by p < 0.05. MAP_90_: monophasic action potential duration at 90% repolarization level.

The time courses of changes in the electrocardiogram variables, atrio-His (AH) and His-ventricular (HV) intervals are summarised in Fig. 2. The pre-drug control values (C) of the PR interval, QRS width, QT interval, QTcV, AH and HV intervals were 109 ± 12, 67 ± 2, 298 ± 22, 326 ± 14, 79 ± 10, and 31 ± 1 ms, respectively. Lapatinib significantly increased the QT interval for 10–15 and 30–60 min and QTcV for 10–60 min after the high dose, respectively. No significant changes were detected in the other variables.

### Effects on the MAP duration during the ventricular pacing, effective refractory period and terminal repolarization period

The time courses of changes in the MAP_90_ during sinus rhythm and the ventricular pacing at a cycle length of 300 and 400 ms, effective refractory period and terminal repolarization period during the ventricular pacing are summarised in Fig. 2. The pre-drug control values (C) of the MAP_90(sinus)_, MAP_90(CL300)_, MAP_90(CL400)_, effective refractory period, and terminal repolarization period were 269 ± 12, 225 ± 8, 248 ± 9, 229 ± 10, and 19 ± 2 ms, respectively. Lapatinib significantly increased the MAP_90(sinus)_ at 60 min, the MAP_90(CL300)_ at 15 and 60 min and effective refractory period for 5–60 min after the high dose, respectively. No significant changes were detected in the other variables.

### Effects on the variables of echocardiogram

The effects of lapatinib on the variables of echocardiogram are summarised in Table 1. No significant change was detected in all variables.

**Table 1:** The effects of lapatinib on the variables of the echocardiogram and left ventricular pressure.

	Cardiac cycle	Variables		Control	0.3 mg/kg	3 mg/kg		P value
					30 min	30 min	60 min	
Dimensions	End-diastole	LVDd	(mm)	25 ± 1	24 ± 1	23 ± 1	24 ± 1	0.54
		LVEDV	(mL)	22 ± 3	21 ± 2	19 ± 2	20 ± 2	0.52
	End-systole	LVDs	(mm)	17 ± 1	16 ± 1	16 ± 1	16 ± 1	0.39
		LVESV	(mL)	9 ± 1	8 ± 1	7 ± 1	9 ± 2	0.24
Systolic function	Ejection period	EF (Teich)	(%)	62 ± 2	61 ± 2	63 ± 3	62 ± 2	0.18
		%FS	(%)	31 ± 2	31 ± 1	33 ± 3	32 ± 2	0.27
Diastolic function	Passive filling	E/A		1.27 ± 0.22	1.28 ± 0.25	1.29 ± 0.27	1.10 ± 0.19	0.52
		E′	(m/s)	0.064 ± 0.011	0.064 ± 0.013	0.060 ± 0.013	0.064 ± 0.012	0.87
		E/E′		7.77 ± 0.80	7.70 ± 0.79	8.45 ± 0.96	7.25 ± 0.50	0.42
		EDPVR	(mmHg/mL)	0.46 ± 0.07	0.59 ± 0.08	0.68 ± 0.19	0.72 ± 0.17	0.14
	Active relaxation	IRT	(ms)	89.9 ± 6.6	93.0 ± 8.6	99.7 ± 9.7	114.5 ± 6.8	0.17

Data are presented as mean ± SEM (n = 5). P values represent the statistical significances within a parameter evaluated with one-way repeated-measures analysis of variance. LVDd: Left ventricular end-diastolic diameter; LVEDV: left ventricular end-diastolic volume; LVDs: left ventricular end-systolic diameter; LVESV: left ventricular end-systolic volume; EF (Teich): ejection fraction assessed by Teichholz method; %FS: % fractional shortening; E/A: the peak velocities of E-wave/A-wave; E′: the peak velocity of E′-wave; E/E′: the peak velocities of E-wave/E′-wave; EDPVR: end-diastolic pressure-volume relationship = left ventricular end-diastolic pressure/LVEDV; and IRT: isovolumic relaxation time.

### Laboratory analysis

**Figure 3:**
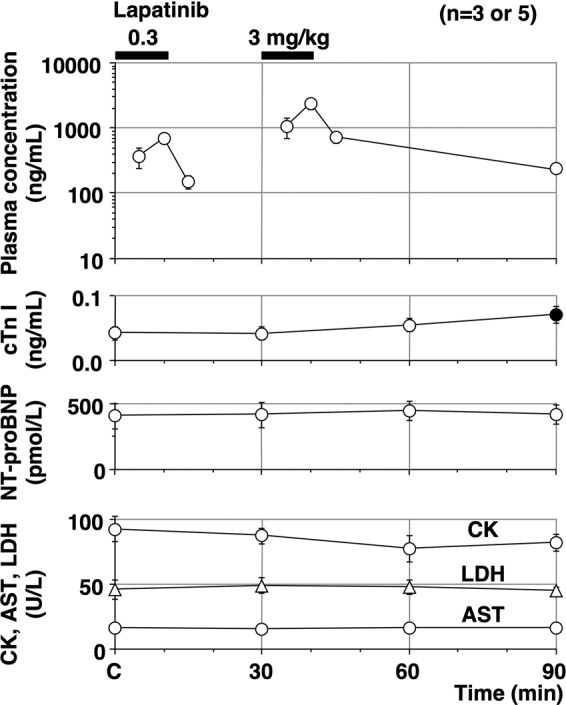
The time courses of the plasma concentrations of lapatinib, cardiac troponin I (cTnI), NT-proBNP, creatinine kinase (CK; circles), aspartate aminotransferase (AST; triangles) and lactate dehydrogenase (LDH; circles). Number of data in cTnI, CK, AST and LDH was 5 besides NT-proBNP. Number of data in NT-proBNP was 3, as the remaining 2 were the lower limit of quantitation during the experiment. Data are presented as mean ± SEM (n = 5). Closed symbols represent significant differences from the corresponding pre-drug basal control values (C) by p < 0.05.

The time courses of the plasma concentrations of lapatinib, cTnI, N-terminal pro-B-type natriuretic peptide (NT-proBNP), creatinine kinase (CK), aspartate aminotransferase (AST), and lactate dehydrogenase (LDH) are summarised in Fig. 3 (n = 3 for NT-proBNP or 5 for the others). The peak plasma concentrations of lapatinib were observed at 10 min after the start of 0.3 and 3 mg/kg infusion, which were 697 ± 73 and 2,358 ± 424 ng/mL, respectively. The pre-control values (C) of cTnI, NT-proBNP, CK, AST, and LDH were 0.042 ± 0.010 ng/mL, 402 ± 92 pmol/L, 92.8 ± 9.5, 16.2 ± 0.7, and 46.2 ± 7.3 U/L, respectively. Lapatinib significantly increased the cTnI at 60 min after the high dose, whereas no significant increase was detected in the other markers.

### Experiment 2: Effects of lapatinib on the chronic atrioventricular block dogs

No electrocardiogram waveform changes or lethal ventricular tachyarrhythmias were observed during the experimental period.

### Effects on the electrocardiogram variables

**Figure 4:**
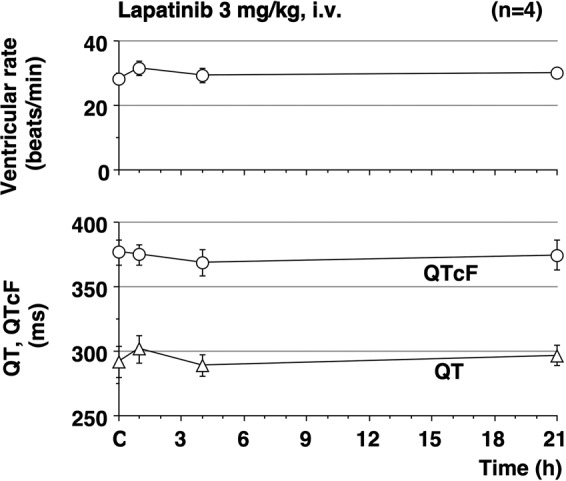
Time courses of changes in the ventricular rate, QT interval, and QTcF after the 3 mg/kg of intravenous administration of lapatinib in the chronic atrioventricular block dogs. Data are presented as mean ± SEM (n = 4).

The time courses of the changes in the electrocardiogram variables and the number of surviving animals are summarised in Fig. 4. The pre-drug control values (C) of the ventricular rate, QT interval, and QTcF were 28 ± 2 beats/min, 376 ± 10 ms, and 292 ± 12, respectively. After the administration of lapatinib, no significant change was detected in these features. Furthermore, no onset of torsade de pointes was observed.

### Beat-to-beat analysis

**Figure 5:**
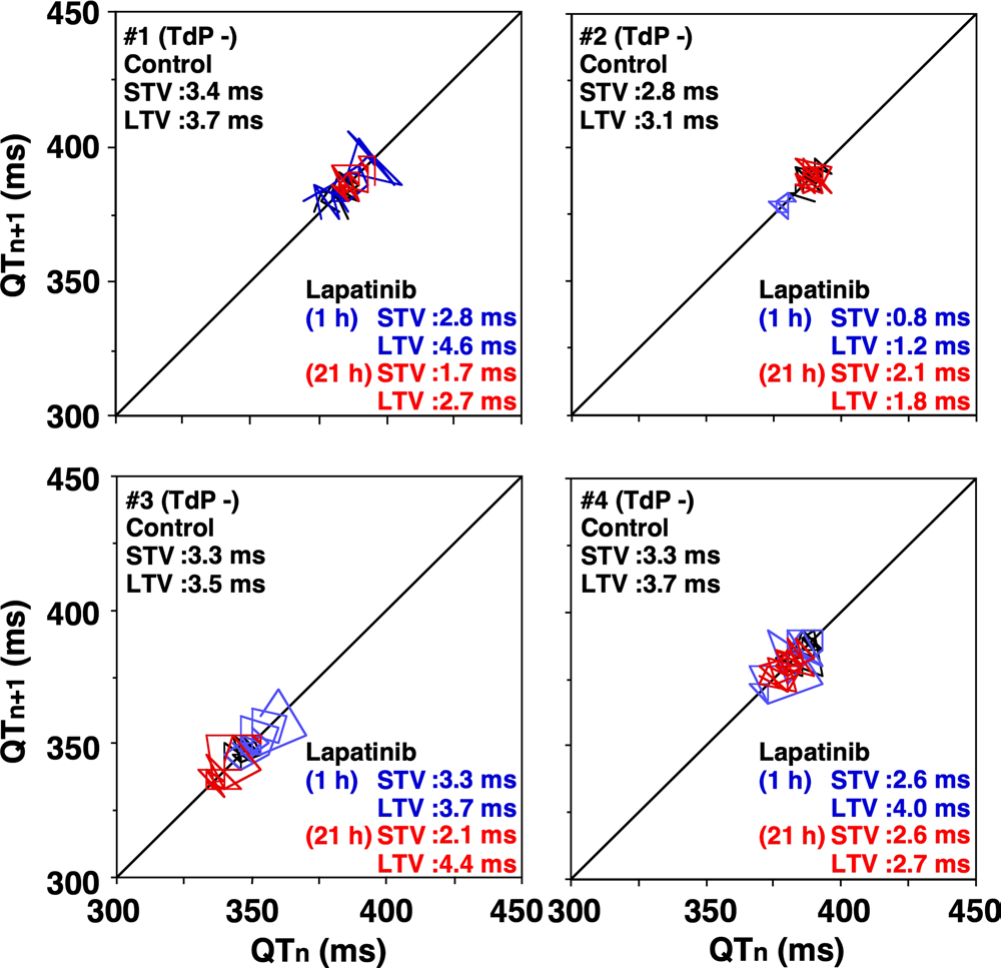
Poincaré plots of the QT interval obtained in each chronic atrioventricular block animal. A total of 31 beats were plotted for each of the two-analysis time-points; namely, before (Pre, black), and 1(blue) and 21 h (red) after the 3 mg/kg of intravenous administration of lapatinib. STV, short-term variability; LTV, long-term variability.

Beat-to-beat analysis was performed in each animal to assess the extent of torsadogenic potential of lapatinib, as depicted in Fig. 5. The QT interval of 31 consecutive beats under stable idioventricular rhythm was measured in each animal before and at 1 and 21 h after the administration of lapatinib. The basal control values of interval in the short-term variability and long-term variability were 2.8–3.4 and 3.1–3.7 ms, respectively. After the administration of lapatinib, no changes were observed in each animal.

## Discussion

Contrary to expectations based on previously reported canine telemetric studies, the therapeutic dose of lapatinib significantly increased the QT interval, QTcV, MAP_90(sinus)_, MAP_90(CL400)_, effective refractory period, but minimally altered the terminal repolarization period and beat-to-beat variability, suggesting that the proarrhythmic potential of lapatinib might not be serious to induce lethal ventricular arrhythmias.

The clinically recommended oral daily dose of lapatinib is 1,250 mg, producing a steady state geometric mean (95% confidence interval) C_max_ value of 2,430 (1,570 to 3,770) ng/mL^9^. In addition, the value was approximately 2.4- to 3.0-fold higher when administered with food^4^. In the present study, lapatinib was intravenously administered in doses of 0.3 and 3 mg/kg over 10 min, providing the peak plasma concentrations of 697 ± 73 (1.2 µmol/L) for the low dose and 2,358 ± 424 ng/mL (4.1 µmol/L) for the high dose, respectively. Since the protein binding ratio of lapatinib to albumin and alpha-1 acid glycoprotein is greater than 99%, its free form concentrations can be roughly calculated as 6.97 and 23.58 ng/mL (12 and 41 nmol/L), respectively^9^. Reportedly, lapatinib inhibits the ErbB1 and ErbB2 receptors, the IC_50_ of which values were 9.2 and 10.8 nmol/L^4^. ErbB4 was inhibited with an IC_50_ of 360 nmol/L (36-fold higher than for ErbB2) and c-Src, a non-receptor tyrosine kinase, was inhibited with an IC_50_ of 3,500 nmol/L (300-fold higher). IC_50_ values for all other 16 tested enzymes were >1,000-fold higher than for ErbB1/ErbB2^4^. Moreover, lapatinib has been reported to slightly inhibit *I*_Ks_ at 3,000 nmol/L, but hardly affect the *I*_Na_, *I*_Ca_ and *I*_K1_ at this concentration^8^. The IC_25_ and IC_50_ values of the hERG current were reported to be 181 and 800 nmol/L, respectively^4,8^. Therefore, the doses of lapatinib used in this study would attain infra- to iso-therapeutic levels of the free plasma concentrations, which can inhibit the ErbB1 and ErbB2 receptors, but may hardly inhibit other protein tyrosine kinases, *I*_Ks_, *I*_Na_, *I*_Ca_ and *I*_K1_, although the hERG current can be modestly inhibited.

Lapatinib did not alter the heart rate, mean blood pressure, cardiac output, peak + d*P*/d*t*, peak −d*P*/d*t*, or the left ventricular end-diastolic pressures, whereas an increased total peripheral vascular resistance was observed at clinically equivalent concentrations. Three months of anti-ErbB2 treatment in humans have been reported to increase systolic and diastolic blood pressure, and plasma concentration of noradrenaline, whereas no change was observed in adrenaline or heart rate^10^. Furthermore, lapatinib has been known to slightly increase the blood pressure in telemetered dogs at the single oral doses of ≥150 mg/kg^4^. The inhibition of ErbB2 in the rostral ventrolateral medulla has been reported to induce hypertension, at least in part, by reducing NO synthesis and inhibiting γ-aminobutyric acid activity in rats^11^. Lapatinib reportedly distributes in the brain at about one-tenth the concentration of blood in rats^12^. In our present study, the concentrations of lapatinib in the brain could be estimated to overcome its IC_50_ values of ErbB2. The high dose of lapatinib increased the total peripheral vascular resistance, suggesting the centrally regulation of vascular tone via inhibition of the ErbB2 receptor. The continuous enhancement of the resistance could increase the strain on cardiac outputs, which might be a risk factor for inducing heart failure especially in the patients after treatments of anthracyclines or trastuzumab treatment.

Lapatinib prolonged the QT/QTcV interval, MAP_90(sinus)_, MAP_90(CL400)_ and effective refractory period after the high dose, but did not alter the PR interval, QRS width or MAP_90(CL300)_. Since the cycle length of the sinus rate was about 600 ms in this study, the prolongation patterns in each MAP_90_ were likely the reverse use-dependent manner, indicating that lapatinib might inhibit the hERG current. In the present study, the continuous prolongation of QT/QTcV interval and the late onset of prolongation of MAP_90(sinus)_ and MAP_90(CL400)_ could be partly explained by these previous findings^7,8^. Our suggestion might be pharmacokinetically supported by the previous report that lapatinib distributed to heart more than blood and decreased from heart slower than blood^12^. Diarrhoea is one of the most common and crucial adverse events of lapatinib, leading to electrolyte imbalances^4,5^. As hypokalemia may contribute to prolong QT/QTcV interval, attention should be paid to the proarrhythmic potential during lapatinib-induced diarrhoea.

Furthermore, lapatinib is considerably less cardiotoxic than trastuzumab, but has been known to demonstrate symptomatic heart failure (0.2%) and asymptomatic cardiac events (1.4%)^13^. Sunitinib, a multi-targeted tyrosine kinase inhibitor, has been reported to induce systolic dysfunction in patients with an incidence of 3–8%^14^. In our previous non-clinical study, it significantly increased the end-diastolic pressure-volume relationship and prolonged the isovolumic relaxation time, in addition to decreasing the amplitude of the peak −d*P*/d*t*^14^. However, lapatinib minimally altered the echocardiogram variables in addition to peak + d*P*/d*t* and peak −d*P*/d*t*, suggesting that its acute cardiotoxicity potential for cardiac contractile performance might be relatively low.

Lapatinib increased the plasma concentration of cTnI without altering NT-proBNP, CK, AST, and LDH. Reportedly, NT-proBNP is a biomarker for reflecting left ventricular wall stress more closely than other ventricular parameters such as left ventricular (LV) ejection fraction, E/e’, and LV longitudinal strain in heart failure^15^. Lapatinib hardly altered the echocardiogram variables and NT-proBNP, suggesting that lapatinib may not damage the LV systolic and diastolic function during the acute phase. cTnI has known to act on myocardial contraction by regulating the calcium-dependent interaction of actin and myosin^16,17^. Although the mechanisms by which cTnI is released into the bloodstream are not fully understood, it is a more sensitive blood biochemical markers for cardiac injuries than CK, AST, and LDH^16,17^. It has been reported that the elevation of cTnI has been observed with the repeated administration of lapatinib combined with paclitaxel or trastuzumab^18^. Although the elevation of cTnI plasma concentration did not robustly predict chronic heart failure in the patients, it likely preceded changes in the LV ejection fraction^18^. It is noteworthy that the intravenous administration of lapatinib acutely increased the plasma concentration of cTnI in our study. Thus, close monitoring of cTnI may become a reliable marker in lapatinib-induced cardiotoxicity.

Lapatinib prolonged the QT/QTcV interval, MAP_90(sinus)_, MAP_90(CL400)_ and effective refractory period after the high dose in the halothane-anaesthetised model (Experiment 1). On the contrary, lapatinib hardly altered QTcF, long-term variability and short-term variability of repolarization in the chronic atrioventricular block dogs (Experiment 2). These discrepancies might be explained the reduced repolarization reserve due to halothane, through suppression of a slow component of the delayed rectifier K^+^ currents together with a rapid component one in the heart and by attenuating the autonomic tone^19^. Thus, these results suggest that lapatinib might not induce lethal ventricular arrhythmias.

In conclusion, the present precise safety pharmacology studies suggest that the therapeutic concentration of lapatinib prolonged QT interval and effective refractory period but did not induce torsade de pointes. These electrophysiological effects in our study may be well correlated with the clinical adverse effects of lapatinib, whereas the acute increase in the total peripheral vascular resistance might provide new insights in lapatinib-induced cardiotoxicity or its mechanism of action. Although the cardiotoxicity might not be so serious, careful monitoring of plasma cTnI concentration, in addition to an electrocardiogram could be a supportive biomarker to predict the onset of lapatinib-induced cardiovascular adverse events.

### Limitations

In our present study, we observed three limitations. First, we did not assess the effects of repeated lapatinib administration on the cardiovascular systems. The lack of the impaired the diastolic function following the single administration of lapatinib dose not confirm the functional safety with repeated administration. Second, we did not assess the effects of lapatinib combined with anthracyclines, paclitaxel or trastuzumab, which are well known to possess cardiotoxicities. They could enhance or expose the toxicities of lapatinib. Third, we did not follow the plasma concentration of cTnI, NT-proBNP, CK, AST, or LDH after our experiment. Although cTnI was the most sensitive blood biochemical marker of the five to predict lapatinib-induced acute cardiotoxicities, we do not negate the possibility of the remaining markers to predict the delayed and/or chronic ones.

## Methods

### Ethics

These experiments were conducted at Faculty of Medicine, Toho University, where all of the authors concurrently belonged to from 2016 to 2018. All experiments were approved by the Toho University Animal Care and User Committee (Nos. 15-55-151, 15-52-272, 16-51-324, 16-53-272) and performed in accordance with the Guidelines for the Care and Use of Laboratory Animals of Toho University. The experiments were also in accordance with the Guidance for the Animals Experiment of the Japanese Society of Toxicology and the Japanese Pharmacological Society.

Experiments were performed using 5 female beagle dogs weighing approximately 10 kg. The animals were obtained through Kitayama Labes Co., Ltd. (Nagano, Japan). The dogs were housed in individual cages on a 12 h light (6:00–18:00)-dark (18:00–6:00) cycle. The ventilation provided a total air exchange rate of 10–15 times per hour. The room temperature was maintained at 23 ± 2 °C, and relative humidity was 50 ± 30%. Each dog was fed with 200 g/day of standard diet (CD-5M; CLEA Japan, Inc., Tokyo, Japan), and was allowed free access to tap water.

### Experiment 1: Effects of lapatinib on the halothane-anaesthetised dogs

This experiment was basically carried out according to our previously reported method assessed for sunitinib^14^. The dogs were initially anaesthetised with thiopental sodium (30 mg/kg, i.v.). After intubation with a cuffed endotracheal tube, 1% halothane vaporized with 100% oxygen was inhaled with a volume-limited ventilator (SN-480-3; Shinano Manufacturing Co., Ltd., Tokyo, Japan). Tidal volume and respiratory rate were set at 20 mL/kg and 15 breaths/min, respectively.

### Cardiohemodynamic variables

Four clinically available catheter sheaths (FAST-CATH^®^ 4061119; St. Jude Medical Daig Division, Inc., Minnetonka, MN, USA) were inserted into the aorta through the right and left femoral arteries and the others were inserted into the inferior vena cava through the right and left femoral veins, respectively. To prevent blood clotting, heparin calcium (100 IU/kg) was intravenously administered through a flush line of the catheter sheath placed at the right femoral vein. A pig-tail catheter was placed at the left ventricle through the right femoral artery to measure the LV pressure, whereas the aortic pressure was measured at a space between the inside of the catheter sheath and outside of the pig-tail catheter through a flush line. The maximum upstroke and downstroke velocities of the LV pressure (peak + d*P*/d*t* and peak −d*P*/d*t*, respectively) were obtained during sinus rhythm to estimate its isovolumic contraction and active relaxation, respectively. In addition, the LV end-systolic and end-diastolic pressures were also measured during sinus rhythm, since the latter reflects the extent of the passive stiffness of the left ventricle^20^. A thermodilution catheter (132F5; Edwards Lifesciences, Irvine, CA, USA) was positioned at the right side of the heart through the right femoral vein. The cardiac output was measured with a standard thermodilution method by using a cardiac output computer (MFC-1100, Nihon Kohden Corporation, Tokyo, Japan). The total peripheral vascular resistance was calculated using the basic equation: total peripheral vascular resistance = mean blood pressure/cardiac output.

### Electrophysiological variables

The surface lead II electrocardiogram was obtained from the limb electrodes. Corrected QT interval (QTcV) was calculated with Van de Water’s formula: QTcV = QT−0.087 × (RR−1000)^21^. A standard 6 French quadpolar electrodes catheter (Cordis-Webster Inc., Baldwin Park, CA, USA) was positioned at the non-coronary cusp of the aortic valve through the left femoral artery to obtain the His-bundle electrogram. A bidirectional steerable monophasic action potential (MAP) recording/pacing combination catheter (1675P; EP Technologies, Inc., Sunnyvale, CA, USA) was positioned at the endocardium of the right ventricle through the left femoral vein to obtain MAP signals. The signals were amplified with a DC preamplifier (model 300; EP Technologies, Inc.). The duration of the MAP signals was measured as an interval, along a horizontal line corresponding to the diastolic baseline, from the MAP upstroke to the desired repolarization level. The duration (ms) at 90% repolarization level was defined as MAP_90_. The heart was electrically driven by using a cardiac stimulator (SEC-3102; Nihon Kohden Corporation) with the pacing electrodes of the combination catheter placed in the right ventricle. The stimulation pulses were rectangular in shape, 1–2 V of amplitude (approximately twice the threshold voltage), and of 1-ms duration. The MAP_90_ was measured during sinus rhythm (MAP_90(sinus)_) and at a pacing cycle length of 400 ms (MAP_90(CL400)_) and 300 ms (MAP_90(CL300)_). The ventricular effective refractory period was assessed with the programmed electrical stimulation. The pacing protocol on the assessment of the ventricular effective refractory period consisted of 5 beats of basic stimuli at a cycle length of 400 ms followed by an extra stimulus of various coupling intervals. Starting in late diastole, the coupling interval was shortened in 5 ms decrements until refractoriness occurred. Starting in late diastole, the coupling interval was shortened in 5 ms decrements until the additional stimulus could no longer elicit a response. The ventricular effective refractory period was defined as the shortest coupling interval that could produce a response. The duration of the terminal repolarization period of the ventricle, reflecting phase 3 repolarization of the action potential, was calculated by the difference between the MAP_90_ and ventricular effective refractory period at the same site, reflecting the extent of electrical vulnerability of the ventricular muscle^14,19–22^.

### Echocardiographic analysis

Two cardiologists certified by the Japanese Circulation Society performed the echocardiographic examinations by using the ultrasound system (GE Vivid i; GE Healthcare Japan, Tokyo, Japan), equipped with a 2.7–8.0 MHz of wide-band frequency-fusion phase-array transducer. The LV end-diastolic diameter, end-systolic diameter, ejection fraction and fractional shortening were measured with the two-dimensional guided M-mode echocardiogram^23^. Estimated LV volume was calculated with the Teichholz formula: LV volume = 7 × (diameter)^3^/(2.4 + diameter)^23^. Ejection fraction and fractional shortening were calculated with following equations: ejection fraction = (LV end-diastolic volume − LV end-systolic volume)/LV end-diastolic volume × 100, and fractional shortening = (LV end-diastolic diameter − LV end-systolic diameter)/LV end-diastolic diameter × 100, respectively^24^. The LV end-systolic pressure-volume and end-diastolic pressure-volume relationships were calculated using the following equations: end-systolic pressure-volume relationship = LV end-systolic pressure/LV end-systolic volume, and end-diastolic pressure-volume relationship = LV end-diastolic pressure/LV end-diastolic volume, respectively. The peak velocities of early diastolic filling wave (E-wave) and atrial contraction wave (A-wave), in addition to the isovolumic relaxation time of LV, were measured with the recordings of transmitral flow velocity pattern. The peak velocity of early diastolic myocardial wave (E′-wave) was measured at the lateral mitral annulus with the tissue Doppler imaging, since diastolic dysfunction was reported to be firstly observed in the lateral wall^25^. The E/A and E/E′ ratios were calculated with the peak velocities of E-wave, A-wave and E′-wave.

### Experimental protocol

The aortic and LV pressures, electrocardiogram, His bundle electrogram and MAP signals were monitored by using a polygraph system (RM-6000, Nihon Kohden Corporation) and analysed with a real-time fully automatic data analysis system (WinVAS3 ver. 1.1R24v; Physio-Tech Co., Ltd., Tokyo, Japan). Each electrocardiogram measurement, MAP as well as AH and HV intervals were determined based on the mean of three recordings of consecutive complexes. The cardiovascular variables were assessed in the following order. The electrocardiogram, His bundle electrogram, aortic and LV pressures and MAP signals were recorded during sinus rhythm. Next, the cardiac output was measured three times. Then, MAP signals were recorded during the ventricular pacing at a cycle length of 400 and 300 ms. Moreover, the ventricular effective refractory period was measured. All parameters described above were usually obtained within 1 min at each time point. Finally, the echocardiographic variables were obtained.

After the basal control assessment, lapatinib in a low dose of 0.3 mg/kg was intravenously administered over 10 min, and cardiohemodynamic and electrophysiological parameters were assessed at 5, 10, 15, 20, and 30 min after the start of the infusion. Next, lapatinib at the high dose of 3 mg/kg was intravenously administered over 10 min, and the parameters were assessed at 5, 10, 15, 20, 30, 45, and 60 min after the start of the infusion. In addition, the echocardiographic study was performed at 30 min after the start of the low dose infusion, and at 30 and 60 min after the start of the high dose infusion.

### Laboratory analysis

A 2 mL volume of blood was drawn from the femoral artery at 10, 15, and 30 min after the low dose, and 10, 15, 30, and 60 min after the high dose. The blood samples were centrifuged at 1,500 *g* for 15 min at 4 °C to obtain the plasma and stored at −80 °C to determine the plasma concentrations of lapatinib, cTnI, NT-proBNP, CK, AST, and LDH. The plasma concentration of lapatinib at 5, 10, and 15 min after the low dose, and 5, 10, 15, and 60 min after the high dose was measured by high-performance liquid chromatographic method followed by tandem mass spectrometry^26^ at Sumika Chemical Analysis Service, Ltd. (Osaka, Japan). The blood biochemical markers were assayed at 30 min after the low dose, and 30 and 60 min after the high dose at LSI Medience Corporation (Tokyo, Japan) besides NT-proBNP. The plasma concentrations of cTnI were measured using a chemiluminescent micro-particle immunoassay, for which the lower detection limit was 0.02 ng/mL, calibration range was up to 50 mg/mL, and analytical sensitivity was 0.02 ng/mL at the 95% level of confidence in humans as well as dogs. NT-proBNP was assayed using Cardiopet^®^ proBNP, for which the detection range was between 250 and 10,000 pmol/L, at IDEXX Laboratories, Inc. (Tokyo, Japan). Data were used from 3 animals for the statistical analysis of NT-proBNP, as two-fifth were the lower limit of quantitation during the experiment.

### Experiment 2: Effects of lapatinib on the chronic atrioventricular block dogs

This experiment was performed in accordance with our previously reported method^27^ and the catheter ablation technique for the atrioventricular node was used as previously described^28^. The dogs were anaesthetised with thiopental sodium (30 mg/kg, i.v.) (n = 4). After intubation with a cuffed endotracheal tube, 100% oxygen was inhaled with a volume-limited ventilator (SN- 480-3; Shinano Manufacturing Co., Ltd.). Tidal volume and respiratory rate were set at 20 mL/kg and 15 strokes/min, respectively. To prevent blood clotting, heparin calcium (100 IU/kg, i.v.) was administered.

### Production of complete atrioventricular block

The surface lead II electrocardiogram was continuously monitored with a polygraph system (RM-6000; Nihon-Kohden Corporation). A quadpolar electrodes catheter with a large tip of 4 mm (D7-DL-252; Cordis-Webster Inc.) was inserted through the right femoral vein using the standard percutaneous technique under the sterile conditions and positioned around the tricuspid valve, observing the bipolar electrograms from the distal electrodes pair. The optimal site for the atrioventricular node ablation was based on the intracardiac electrogram, of which a very small His deflection was recorded, and the atrial/ventricular voltage ratio was > 2. The site was usually occurred at 1–2 cm proximal from the position, where the largest His bundle electrogram was recorded. The power source for atrioventricular node ablation was obtained using an electrosurgical generator (MS-1500; Senco Medical Instrument Manufacturing Co., Ltd., Tokyo, Japan), which delivers continuous unmodulated radiofrequency energy at a frequency of 500 kHz. After proper positioning, the radiofrequency energy of 20 W was delivered for 10 s, from the tip electrode to an indifferent patch electrode positioned on the animal’s back, which continued for 30 s if junctional rhythm was induced. The endpoint of this procedure was the development of a complete atrioventricular block with the onset of stable idioventricular escaped rhythm.

### Holter recording

A Holter recording and analysis system (QR2100 and HS1000; Fukuda ME Kogyo, Tokyo, Japan) was used to record and analyse the electrocardiogram over 24 h. The effects of lapatinib on the ventricular rate, QT interval, and corrected QT calculated with the Fridericia’s formula: QTcF = QT/(RR/1000)^1/3^, in addition to their proarrhythmic effects, were assessed without anaesthesia. The ventricular rate, QT interval, and QTcF were expressed as the mean of 10 consecutive complexes. In this study, torsade de pointes was defined as a polymorphic ventricular tachycardia associated with QT-interval prolongation, consisting of 5 beats or more twisting QRS complexes around the baseline^29^.

### Experimental protocol

Experiments were conducted at least 4 weeks after the induction of complete atrioventricular block. We have assessed the pro-arrhythmic effects of several drugs with a group size of 4–6, which has sufficient sensitivity and reliability to detect drug-induced torsade de pointes^19^. About 2 h after the start of Holter recording, 3 mg/kg of lapatinib was intravenously infused over 10 min without anaesthesia. The electrocardiogram variables at 1 h before the drug administration were defined as the control, and the electrocardiogram was recorded for >20 h when lethal arrhythmia was not induced.

### Beat-to-beat analysis

An electrocardiogram of 31 consecutive beats under the stable idioventricular automaticity without ectopic activity was adopted before, and at 1 and 21 h after the drug administration. When the QT interval was obscured by the P wave, we estimated the end of T wave by cancelling the component of the P wave from the electrocardiogram waveform on the screen. Poincaré plots with QT_n_ versus QT_n+1_ were prepared for each of three analysis time-points. The mean orthogonal distance from the diagonal to the points of the Poincaré plot was determined as short-term variability (=∑|QT_n+1_ − QT_n_|/[30 × √2]). On the contrary, the mean distance to the mean of the parameter parallel to the diagonal of the Poincaré plot was determined as long-term variability (=∑|QT_n+1_ + QT_n_ − 2QT_mean_|/[30 × √2]). These nomenclatures were adopted from investigations of heart rate variability in human beings^30^, which have been applied to the QT-interval analysis in normal dogs and chronic atrioventricular block dogs^31^.

### Drugs

Lapatinib (Biorbyt Ltd., Cambridge, UK) was dissolved in dimethylsulfoxide of 100 mg/mL (0.1 mg/µL). The solution was injected at a dose of 30 µL/kg to the experimental dogs. The other drugs used were thiopental sodium (Ravonal^®^ 0.5 g for Injection, Mitsubishi-Tanabe Pharma, Osaka, Japan), halothane (Fluothane^®^, Takeda Pharmaceutical Company, Osaka, Japan), heparin calcium (Caprocin^®^, Sawai Pharmaceutical Co., Ltd., Osaka, Japan), benzylpenicillin potassium (Meiji Seika Pharma Co., Ltd., Tokyo, Japan) and streptomycin sulphate (Meiji Seika Pharma).

### Statistical analysis

Data are presented as mean ± standard error of mean (SEM). The statistical significances within a parameter were evaluated by one-way repeated-measures analysis of variance (ANOVA) followed by Contrasts as a post-hoc test for mean values comparison. A p value < 0.05 was considered to be statistically significant.
